# Potential of Stem Cells and CART as a Potential Polytherapy for Small Cell Lung Cancer

**DOI:** 10.3389/fcell.2021.778020

**Published:** 2021-12-03

**Authors:** Evgenii Skurikhin, Olga Pershina, Mariia Zhukova, Darius Widera, Natalia Ermakova, Edgar Pan, Angelina Pakhomova, Sergey Morozov, Aslan Kubatiev, Alexander Dygai

**Affiliations:** ^1^ Laboratory of Regenerative Pharmacology, Goldberg ED Research Institute of Pharmacology and Regenerative Medicine, Tomsk National Research Medical Centre of the Russian Academy of Sciences, Tomsk, Russia; ^2^ Stem Cell Biology and Regenerative Medicine Group, School of Pharmacy, University of Reading, Reading, United Kingdom; ^3^ Institute of General Pathology and Pathophysiology, Moscow, Russia

**Keywords:** small cell lung cancer, COPD, cancer stem cell, inflammation, CART therapy

## Abstract

Despite the increasing urgency of the problem of treating small cell lung cancer (SCLC), information on the causes of its development is fragmentary. There is no complete understanding of the features of antitumor immunity and the role of the microenvironment in the development of SCLC resistance. This impedes the development of new methods for the diagnosis and treatment of SCLC. Lung cancer and chronic obstructive pulmonary disease (COPD) have common pathogenetic factors. COPD is a risk factor for lung cancer including SCLC. Therefore, the search for effective approaches to prevention, diagnosis, and treatment of SCLC in patients with COPD is an urgent task. This review provides information on the etiology and pathogenesis of SCLC, analyses the effectiveness of current treatment options, and critically evaluates the potential of chimeric antigen receptor T cells therapy (CART therapy) in SCLC. Moreover, we discuss potential links between lung cancer and COPD and the role of endothelium in the development of COPD. Finally, we propose a new approach for increasing the efficacy of CART therapy in SCLC.

## Introduction

SCLC accounts for 10–15% of all known cases of lung cancer and belongs to the most malignant tumors (Bray et al., 2018; Walcher et al., 2020). SCLC is characterized by its asymptomatic and rapid progression, as well as by early metastasis. The highest incidence of SCLC is recorded in the 40-60 age group. All types of small cell carcinoma have a poor prognosis even in cases when the diagnosis is made at an early stage (Bray et al., 2018; Walcher et al., 2020). In the vast majority of cases, SCLC develops in smokers, with men having a higher incidence. However, lung cancer is also a serious complication in patients with COPD and accounts for approximately 15% of deaths in patients with COPD (Sekine et al., 2012; Denholm et al., 2014).

Standard treatment for diagnosed SCLC is chemotherapy based on platinum preparations with etoposide and radiation therapy (Wang et al., 2019). Patients whose tumor recurs more than 6 months after fist-line chemotherapy are re-treated using the same regimen. Topotecan, with a response rate of 15–20% and an overall survival rate of 1 year in 30% of cases is a second-line treatment for SCLC. The options for subsequent lines of treatment are limited. CART therapy is a promising approach to treating tumors. CART therapy uses antibodies against inhibitory signaling molecules expressed on tumor cells and immune system cells (Dotti et al., 2014). T-cells modified by chimeric antigen receptors have been shown to be effective in patients with hematological malignant neoplasms (Schubert et al., 2016). In addition, safety and applicability of CART therapy has been confirmed for solid tumors. However, there are no reported clinical trials using CART-cells for the treatment of SCLC (Dotti et al., 2014). This is largely due to the high mutational activity of the tumor. Thus, applicability of known therapeutic approaches to SCLC treatment are rather limited, highlighting the urgent need for new treatment methods.

In this review, we analyzed existing reports on diagnosis and treatment of SCLC and propose a novel potential approach to its treatment.

## Small Cell Lung Cancer

### Epidemiology

Lung cancer is the most common cancer and the leading cause of cancer-related death in both men and women (Bray et al., 2018; Walcher et al., 2020). There are approximately 2.1 million cases of lung cancer and about 1.7 million deaths worldwide each year. Lung cancer claims more lives each year than breast, prostate, and colon cancers combined. In most cases, lung cancer originates from epithelial tissue of the bronchi of various sizes. It can be central, peripheral, or massive, that is, mixed, depending on the origin. WHO identifies two main histological types of lung cancer—non-small cell lung cancer (NSCLC) and small cell lung cancer (SCLC). NSCLC occurs in 85% of all lung cancer cases (Oser et al., 2015; Zamay et al., 2017) and includes squamous cell lung cancer, which originates in the main bronchi (25–30%), adenocarcinoma (40% of cancers) occurring in the small bronchi, and large cell carcinoma (10%) (Lemjabbar-Alaoui et al., 2015). SCLC accounts for 15% of lung cancer cases, and is characterized by an aggressive course. SCLC is believed to be derived from pulmonary neuroendocrine cells (Zamay et al., 2017; Wang et al., 2020). According to the International Association for the Study of Lung Cancer (IASLC), SCLC can be divided into two stages—limited stage and advanced stage. The majority of patients (approximately 70%) with SCLC are diagnosed with advanced stage (Wang et al., 2020). This is at least partly responsible for the poor prognosis.

The overall patients’ survival rate with a limited stage of SCLC is from 15 to 20 months and for patients with an advanced stage of SCLC 8–13 months. The 5-year survival rate of patients is 10–13% and 1–2%, respectively (Horn et al., 2016).

Smoking is a major risk factor for developing SCLC. Other risk factors include exposure to asbestos and other carcinogens, such as beryllium, cadmium, silicon dioxide, chlorine compounds, other chemicals, and general air pollution. In addition, hereditary factors play a role if there is a history of lung cancer in close relatives (Wang et al., 2020). In SCLC, several mutations have been found that lead to inactivation of tumor suppressor genes. These include tumor protein 53 (TP53) (75–90%) (Wistuba et al., 2001), retinoblastoma 1(RB1) (60–90%) (Mori et al., 1990; Arriola et al., 2008), and phosphatase and tensin homolog (PTEN) (2–4%) (Kim et al., 1998). Moreover, activation mutations of the oncogenes phosphatidylinositol-4,5-bisphosphate 3-kinase (PIK3CA), epidermal growth factor receptor (EGFR), and v-Ki-ras2 Kirsten rat sarcoma viral oncogene homolog (KRAS) are often found in SCLC (Tatematsu et al., 2008; Shibata et al., 2009). In addition, a change in the expression of the MYC gene family, amplification of the EGFR/BCL2 and a deficiency of the RASSFIA/PTEN/Fhit were observed (Onuki et al., 1999).

### Small Cell Lung Cancer Treatment

Until the 1970s, SCLC was not considered an independent nosological unit, and the results of treatment were assessed without taking into account the histological type of lung cancer. Classification of SCLC as a separate disease made it possible to assess the effectiveness of adriamycin-containing treatment regimens, such as CAV ((C)yclophosphamide, (A)driamycin, (V)incristine) (Tashiro et al., 1989; Walcher et al., 2020). In the early 1980s, combinations with nitrosourea derivatives began to be prescribed for patients with SCLC. A little later, etoposide was prescribed as an additional treatment option. In the 1990s, treatment regimens including taxanes and topoisomerase I inhibitors were introduced (Karim and Zekri, 2012).

Currently, there are three main approaches to the treatment of SCLC: surgery, radiation therapy, and chemotherapy. Chemotherapy is central to the treatment of SCLC. Front-line drugs are platinum preparations (cisplatin or carboplatin) in combination with etoposide. With an extensive stage of tumor progression, drugs such as doxorubicin, cyclophosphamide, vincristine, and others are prescribed (European Society for Medical Oncology, 2021; www.esmo.org; www.cancer.gov/types/lung/hp/small-cell-lung-treatment-pdq).

Although patients with SCLC initially respond well to cytotoxic therapy, tumor recurrence occurs in most patients (Giaccone et al., 1987). Patients whose tumor recurs more than 6 months after fist-line chemotherapy should be re-treated using the original treatment regimen. Additionally, topotecan has been introduced into SCLC chemotherapy (Eckardt et al., 2007). Topotecan is characterized by a high response rate (15–20%) and an overall survival rate of 30% within 1 year. Currently, it is a second-line therapy for SCLC.

Most of the clinical observations indicate a low effectiveness of subsequent lines of treatment. With second-line chemotherapy, the 5-year survival rate for patients with SCLC is below 5% (Gaspar et al., 2012; Lallo et al., 2019). In 2013, the National Cancer Institute placed SCLC in the category of resistant tumors and identified it as a priority for research. However, the situation has not changed dramatically since this recommendation (Lallo et al., 2019; National Cancer Institute, 2021).

The rapid development of drug resistance complicates the treatment of SCLC (Byers and Rudin, 2015). It has been recognized that cells which make up the tumor microenvironment secrete enzymes, anti-inflammatory cytokines, and chemokines causing thereby changes in tumor and immune cells contributing to the escape from immunological surveillance and reduction of the immune response (Lallo et al., 2019). Such mechanisms of tumor activity should be taken into account when developing novel approaches to antitumor therapy.

In the past few years, immunotherapeutic methods have been developed that use or enhance the patient’s own immune system to target tumor cells (Sharma and Allison, 2015; Topalian et al., 2015). At the same time, cancer immunotherapy has developed in parallel with the improved understanding of the role of the immune system in tumorigenesis. Among the approaches to stimulate the immune response, vaccines, immunomodulators, and monoclonal antibodies (MAB) can be applied. MABs are directed against checkpoint inhibitor on activated T-cells and/or tumor cells. Thanks to immunotherapy, many patients with advanced lung cancer are in long-term remission and have overall longer survival rates. In this context, the IMPOWER-133 trial showed longer progression-free survival and overall survival in patients treated with etoposide/carboplatin/atezolizumab (Carbone et al., 2017; Horn et al., 2018). Currently, several clinical trials focused on the efficacy and safety of “immune checkpoint inhibitors” in small cell lung cancer patients are being conducted (NCT01331525; NCT02359019; NCT02538666; NCT02481830; NCT02701400; IMPOWER-133; KEYNOTE-028; CheckMate-032) (Yang et al., 2019). However, clinical application of immune checkpoint inhibitors is still associated with limited response rates, severe immune-related adverse events, development of resistance, and more serious exacerbation of cancer progression referred to as hyper-progressive disease (Agrawal, 2019).

## Chronic Obstructive Pulmonary Disease as a Small Cell Lung Cancer Risk Factor

Patients with diseases of the respiratory system (COPD, chronic bronchitis, pulmonary emphysema) have an increased risk of developing lung cancer. Annually, about 1% of patients with COPD develop lung cancer, and the prevalence of COPD in patients with lung cancer is 8–50% (Sekine et al., 2012). Thus, COPD is an independent risk factor for lung cancer (Denholm et al., 2014). Most studies so far investigated the link between COPD and NSCLC. However, there is little data on the association of COPD with the development of SCLC. This is mainly due to the lower prevalence of SCLC compared to NSCLC (Ju et al., 2018).

COPD and lung cancer are different diseases, but they share similar pathogenetic mechanisms, including epithelial-mesenchymal transition, inflammation, oxidative stress, and DNA damage. In COPD, inflammation is found in the airways and airspace and is inherently destructive (Houghton, 2013). In patients with COPD, chronic inflammation is a key feature that positively correlates with disease severity (Hogg et al., 2004) and may be a potential factor in the development of lung cancer in proximal and distant tissues (Parris et al., 2019). Cells such as neutrophils, macrophages, CD4^+^ and CD8^+^ lymphocytes are involved in the pathogenesis of COPD and lung cancer (Barnes et al., 2003; Paone et al., 2011; Gomes et al., 2014).

Recent reports point to different specific characteristics of immune cells in lung cancer and COPD. In patients with COPD, alveolar macrophages with the M1 phenotype have been found to polarize to the Th1 phenotype via interferon-γ. This cytotoxic Th1 phenotype is favorable for the tumor microenvironment, but does not occur in solid tumors, where immune cells are predominantly represented by the Th2 phenotype, activated by M2 macrophages (Shaykhiev et al., 2009). Changes in the composition of immune cells may be a useful biomarker in COPD patients with a precancerous condition. It has been shown that an increase in Th1-polarized CD4^+^ T cells in the lung tissue is associated with the severity of COPD and may be useful in assessing disease progression (Barnes et al., 2003; Paone et al., 2011; Gomes et al., 2014). On the other hand, these inflammatory cells with specific characteristics act as targets and suggest the possibility of targeted therapy for COPD and, separately, precancerous condition and lung cancer.

Co-expression of PD-1, T-cell immunoglobulin, and molecule-3, containing the mucin domain (TIM-3) on CD8^+^ T-cells, increases with the severity of COPD (Biton et al., 2018). PD-1 blockade is considered as one of the treatment approaches to both reduce the severity of COPD and reduce the likelihood of developing lung cancer. Recent studies have demonstrated an increase in progression-free survival among patients with advanced NSCLC with concomitant COPD receiving anti-PD-1 therapy (Biton et al., 2018; Chalela et al., 2018). The effectiveness of anti-PD-1 therapy among COPD patients is explained by immunological dysregulation leading to increased expression of immune checkpoints among T-cells (Chalela et al., 2018; Mark et al., 2018).

Various approaches to the treatment of lung cancer, including SCLC, have been presented above. The overwhelming majority of treatment options focus on patients with a localized tumor process (limited stage) and a widespread tumor process (advanced stage). This is largely due to the ability to diagnose lung cancer during this period of the development of the disease, including in patients with COPD. Meanwhile, precancerous conditions in patients without and with COPD are practically not covered. This is due to a very fine line of the transition from COPD to SCLC, and due to the lack of approaches to diagnose this process. During the searching for potential biomarkers of the precancerous condition, we turned our attention to such an important pathological process in COPD as systemic endothelial dysfunction (Green and Turner, 2017) with increasing apoptosis of lung endothelial cells (Peinado et al., 2006). An expected result of this is a decrease in alveolar vascularization (Wedzicha et al., 2016). Pulmonary microvascular blood flow and vascular density are already reduced in mild COPD, including in areas of the lungs without overt emphysema (Hueper et al., 2015). In patients with severe COPD, destruction of the microvascular bed is increasing (Hueper et al., 2015). It is known that in patients with pulmonary emphysema, a decrease in the number of endothelial progenitor cells in the blood was noted, and this indicator was the lowest in pan lobular emphysema (Doyle et al., 2017). The results of our own research confirm these data. We propose that damage to the lung endothelium is a factor in the progression of COPD. We can already point out several parameters within the endothelium that can be used as prognostic biomarkers of complications. These include changes of the numbers of endothelial cells, endothelial progenitor cells, pericytes, and smooth muscle cells (Pakhomova et al., 2020). In this regard, angiogenesis can be beneficial for the pulmonary vasculature and alveolar epithelium in pulmonary emphysema and COPD, and thus, presumably, it is possible to prevent the negative development of events: the transition of the disease to a precancerous condition, induction of lung cancer.

## T-Cells in CART Therapy

**FIGURE 1:**
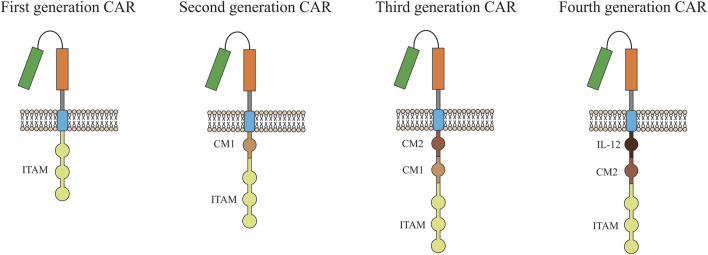
Evolution of chimeric antigen receptor (CAR) from the first generation to the fourth generation. Single chain antibody (CD3ζ or FcεRIγ) links the ITAM at transmembrane region for the first generation. Costimulatory molecule (CM1), such as CD28, has been engineered to the signal transduction region for the second generation. Another costimulatory molecule (CM2) based on the second generation for the third generation has been engineered to the signal transduction region. The interleukin-12 (IL-12) based on the second generation for the fourth generation has been engineered to the signal transduction region. ITAM, Immunoreceptor tyrosine-based activation.

One of the promising methods for eliminating tumor cells may be the use of T-cells with a chimeric antigen receptor (CAR). CARs are modular synthetic receptors with a fragment of a monoclonal antibody designed to selectively bind to specific antigens on the surface of the plasma membrane of tumor cells. The result of the interaction of CAR with a tumor antigen is the activation of T-cell antitumor response (Stern et al., 2020). First generation CARs contained only the CD3ζ, or Fc gamma signaling domains (Figure 1). Over time, technology for generating CART cells has improved significantly, which made it possible to add co-stimulating domains such as CD28, 4-1BB, or OX4. Second-generation CARs have been supplemented with one domain while more than two domains can be added to modern CARs (Srivastava and Riddell, 2018).

As noted above, CART cells are designed to recognize specific tumor antigens and eradicate tumor cells. A CD19-specific CART cell therapy has been shown to be effective in B-cell malignancies, including acute lymphoblastic leukemia (ALL), chronic lymphocytic leukemia (CLL), and non-Hodgkin’s lymphoma (NHL) (Schubert et al., 2016). However, no definite positive results have been achieved in the treatment of solid tumors with CART cells, in particular, due to the difficulties in the selection of T-cells for the formation of CAR, the selection of a molecular and cellular target, and the recruitment of CART cells. These difficulties and limitations have yet to be overcome.

The therapeutic efficacy of CART anti-cancer therapy largely depends on the choice of T-cell subpopulations and the nature of their modification. In particular, CART cells as the final cell product must have the ability to proliferate in order to obtain a cell mass sufficient for anti-cancer therapy (Wang et al., 2018). In CART therapy, attempt shave been made to use various CD4^+^ and CD8^+^ T cells (Golubovskaya and Wu, 2016). It is known that CD4^+^ T-cells mediate systemic immunity, which is important for long-term tumor elimination (Wang et al., 2018). CD8^+^ T-cells have the potential not only to recognize, but also to destroy tumor cells (Gascón et al., 2020). Although following CAR modification, cytotoxic CD8^+^ T-cells mediate the direct eradication of tumor cells, also CD4^+^ T helper cells (Th cells) have been identified as highly efficient and clinically important T-cells (Stock et al., 2019). Recent studies indicate that CD4^+^ CART cells have a cytotoxic ability comparable to cytotoxic CD8^+^ CART cells (Stock et al., 2019), and can increase the proliferation of CD8^+^ T cells (Yang Y. et al., 2017). Based on data inferred from T cell biology, trials have been designed and conducted using defined mixtures of CD8 and CD4 T cells to generate CAR products that have demonstrated success in CD19^+^ leukemias and lymphomas (Gascón et al., 2020).

Cancer immunotherapy can include the use of different subpopulations of memory T-cells and effector T-cells in CART therapy. Subpopulations of CD4^+^ memory cells (Th1, Th2, Th9, Th17, Th22, Treg, and Tfh) and effector CD8^+^ cells differ in many parameters including expression of extracellular (CD25, CD45RO, CD45RA, CCR-7, L-selectin or CD62L, etc.) and intracellular markers (FOXP3), epigenetic and genetic factors, and lastly metabolic pathways (catabolism and anabolism). By influencing these parameters, CART therapy can be improved (Golubovskaya and Wu, 2016). It is believed that during immunotherapy, it is important to maintain and/or create a certain ratio between CD4^+^ Th-cells and cytotoxic CD8^+^ T-cells. The ratio of CD4^+^ T-cells/CD8^+^ T-cells in the cell product can affect the effectiveness of antitumor CART therapy. To resolve this issue, the desired cell subpopulations must be isolated, and CARs need to be separately modified. As a result, it is possible to adjust the ratio of CD4^+^ T cells/CD8^+^ T cells in a patient specific manner (Stock et al., 2019). However, this approach complicates the production process.

The differentiation status of T-cells proposed for modification plays a certain role in achieving a positive effect during CART therapy. There is evidence that transfusion of a large number of undifferentiated CART cells has a beneficial effect on treatment (Stock et al., 2019). Undifferentiated CART cells are characterized by high proliferative activity, high viability *in vivo*, and long-term antitumor activity. The therapeutic characteristics of the cell product are influenced not only by the T-cell construct included in the CAR, but also by the culture conditions during the *ex vivo* expansion of the T cells as well. When obtaining the required cell mass of T-cells, cytokines, and pharmacological inhibitors of specific differentiation pathways are used. Currently a standardized and optimal production process for obtaining CART cells has not yet been established. Thus, defined and effective criteria for predicting the response to CART therapy are absent.

Since blood samples are easier to obtain than a lung biopsy, the study of the antigenic profile (immunophenotype) of immune cells circulating in the blood in order to predict the immune response has recently become an increasingly frequent method. Patients with a good response to checkpoint inhibitors usually have fewer T-cells in the peripheral blood compared to patients who do not respond to therapy (Gascón et al., 2020). The initial ratio of T-cell subpopulations, including the content of poorly differentiated cells, in the blood of patients is reflected in the result of CART therapy. In particular, patients with large numbers of circulating tumor cells (CTCs) may have a small number of poorly differentiated T-cells in their blood. As a result, CTCs can be the preferred target for exogenous cytokines compared to CART cells. In such a situation, the cytotoxic activity of T cells against the tumor will be significantly reduced (Gattinoni et al., 2005). Therefore, to provide additional cytokine therapy during CART therapy, it is desirable to select T-cells for CAR modification in patients with a small number of CTCs in the blood.

Generation of CAR in T-cells begins with the collection of unstimulated leukocytes from the blood using leukapheresis. Isolation of the required cell population from the total fraction of lymphocytes is carried out taking into account their functional and structural features. This is based mainly on the assessment of surface markers CD4, CD8, CD25, or CD62L (Levine, 2015). Isolation of the patient’s autologous antigen-presenting cells (APCs) for subsequent activation requires additional multi-stage manipulations, which make it difficult to obtain active CART cells in the required period of time. In this regard, alternative approaches have been developed to increase the efficiency. These include the use of beads coated with MABs specific to the CD3 and CD28 antigens (Suhoski et al., 2007). During the activation process, cells are incubated with a viral vector, which is used to deliver RNA encoding a chimeric antigen receptor into T-cells, followed by reverse transcription into DNA and insertion into the patient’s T-cell genome. Thereafter, CAR begins to be expressed on the surface of the patient’s T-cells (Montini et al., 2009). The final stage of the protocol for the modification of T-cells *in vitro* is obtaining the required amount for administration to the patient (Gattinoni et al., 2005). Part of the cell material is cryopreserved and, if necessary, thawed to confirm its characteristics and to conduct repeated cell therapy (Levine, 2007).

The resistance of tumors to a number of therapeutic agents is well-known. Resistance is based on the functional and structural heterogeneity of the cellular composition of the tumor. It should be noted that it is possible for a tumor to acquire resistance to subsequent therapy with CART cells and to relapse. After a T-cell therapy with CD19-CAR, 70–90% of patients with B-cell malignant neoplasms developed a stable clinical remission. Subsequent clinical trials showed a loss and/or suppression of CD19 antigen expression in 70% of treated patients, which was considered causative for the occurrence of relapses after treatment (Levine, 2007; Maude et al., 2015).

An ideal situation for CART cell therapy is when the target antigen is overexpressed on all or the vast majority of tumor cells and is not expressed on the surface of healthy cells. However, the majority of tumor-associated antigens (TAA) are expressed on the cell membrane of patients with various types of cancer, including NSCLC and SCLC. Many of them are expressed at high levels and by healthy cells. Thus, CART cells might attack healthy tissue, which can lead to serious side effects (Zhong et al., 2020). Importantly, even a slight expression of tumor associated antigens on the surface of healthy cell membranes leads to damage and dysfunction of the lungs, blood system, and gastrointestinal tract and can be life-threatening (Bonifant et al., 2016). Therefore, to reduce the risk of side effects, CART cells should be as selective as possible. One of the options for increasing selectivity is an appropriate choice of the isoform of the target antigen. Expression of one antigen isoform can be observed on tumor cells, and another isoform on healthy cells. Examples for this strategy are CART therapies targeting EGFRvIII and CD44v8-10 (Morgan et al., 2012; Kim and Ryu, 2017). Improving the recognition of the target antigen by CART cells also allows reducing cellular toxicity to healthy tissues, as well as the incidence of relapses and tumor resistance to CART therapy. These methods are based on the creation of CART cells capable of recognizing several antigens, which significantly increases the specificity and efficiency (Walcher et al., 2020).

Various independent research groups have designed CARs containing two independent domains (Grada et al., 2013; Zah et al., 2016). These CAR T cells work according to the “OR-gate” and can be activated by two antigens and the binding of either antigen induces T-cell activation. One OR-gate strategy utilizes the pooled mixture of two populations of CART cells (CAR pool), each expressing a monospecific CAR. A variation on this theme is to sequentially administer two different CART cell products. Another strategy is the co-expression of two separate CARs in each T cell (dual CAR). Yet another approach uses tandem bispecific CARs (TanCAR) that comprise two scFv domains separated by a linker on one receptor chain, and this strategy was shown to be functionally superior to both the CAR pool and dual-CAR approaches (Hegde et al., 2016; Zah et al., 2020). Under conditions of the developing resistance risk due to a decrease or termination of the expression of the target antigen of the cancer cell, this type of CAR T cell construction can be effective. In particular, CARs targeting two B-cell specific antigens: CD19 and CD22, or CD19 and CD20. These CARs are less sensitive to tumor cell resistance in absence of CD19 (Shah et al., 2019). The disadvantage of this approach is the increased likelihood of a toxic effect in relation to healthy cells and tissues, since with an increase in the number of CAR application points, the number of healthy target cells naturally increases.

Another approach to reduce the toxic effect on healthy cells is to design CAR T cells that work according to the “NOT gate.” Here, a T-cell is engineered to have two specific CARs, one of which is activating and the other is inhibitory (iCAR). The principle of this method is when a T-cell expressing both CAR and iCAR meets a cell expressing only the target CAR antigen, it kills the target, but when both CAR and iCAR antigens are encountered on the same cell, negative iCAR regulatory signaling will predominate or attenuate CAR signaling and reduce the activation of T-cells. This approach is possible due to the fact that iCARs have an extracellular domain of antigen recognition, but their intracellular domains, instead of containing activating domains, carry signaling domains from immune inhibitory receptors such as (programmed cell death 1) PD-1 and cytotoxic T-lymphocyte-associated protein 4 (CTLA-4) (Fedorov et al., 2013). The disadvantage of this approach is that it is impossible to separate healthy cells from tumor cells using a single surface marker.

Another method that improves the accuracy of targeting CART cells to tumor cells is an approach to designing CART cells that work according to the “AND” gate, which requires the simultaneous activation of two receptors for the manifestation of an immune response. In this approach, activation of one receptor by a tumor antigen induces the expression of a second CAR, which triggers the destruction of tumor cells. A similar approach is implemented using the synthetic receptor Notch (synNotch) (Roybal et al., 2016b). SynNotch is a synthetic transmembrane receptor, with a cytoplasmic domain that equipped with a transcriptional regulator that is released from the membrane when the ligand interacts with the receptor. Subsequently, it enters the cell nucleus, thereby activating the transcription of target genes (Morsut et al., 2016). In this scenario, synNotch controls the expression of CAR. This CAR triggers T-cell cytotoxicity and shows significant advantages over CAR T cells that have specificity for only one antigen. In preclinical studies, a higher accuracy of the destruction of tumor cells has been shown. Importantly, healthy tissues of animals expressing only one antigen remained unaffected (Roybal et al., 2016a).

Table 1 shows clinical trials with lung cancer as indication (https://clinicaltrials.gov/) registered as of September 2021 (ClinicalTrials, 2021).

**TABLE 1:** Clinical trials targeting lung cancer (https://clinicaltrials.gov/) registered as of September 2021.

Condition or disease	Number of clinical trials
Small cell lung cancer	1,063
CART cell	581
Chimeric antigen receptor T-cells	671
CART cell lung cancer	16
CART cell immunotherapy	77
Lung cancer chimeric antigen receptor T-cells	18
CART cell immunotherapy lung cancer	4
CART cell immunotherapy CD133 solid tumors	0
CD133 solid tumors	4
CART cell immunotherapy MUC1	2
PD-L1 antibody small cell lung cancer	8
Small cell lung cancer CART cell immunotherapy	0
Small cell lung cancer CTLA-4	5
Cancer stem cells chimeric antigen receptor-modified T cells	3
Cancer stem cells small cell lung cancer	15
Cell lung cancer epidermal growth factor receptor	284
Small cell lung cancer epidermal growth factor receptor	3
CART mesothelin	19
Cell lung cancer CAR T mesothelin	4
SCLC ICI	1 (Phase 3)
SCLC immunotherapy	42
	(34 studies for phase 1, 2;
	4 studies for phase 3;
	no studies for: phase 4)
Small cell lung cancer CART	1 (Phase 1)
Small cell lung cancer CART Mesothelin	0
Cancer stem cells small cell lung cancer CART cell immunotherapy	0

In the overwhelming majority of cases, these studies are focused on therapy of NSCLC. Notably, no clinical trials have been conducted involving CART therapy for the treatment of SCLC. Moreover, there are no clinical trials focusing on adoptive T-cell therapy at the 4 phase. We found only an alone clinical trial using CART-cells for the treatment of SCLC (Phase 1, NCT03392064). Chen et al. demonstrated that Delta-like 3-targeted (DLL3) bispecific antibody plus PD-1 inhibition was effective in controlling SCLC growth (Chen et al., 2020). However, investigators have recognized four major distinct subcategories of SCLC using unbiased RNA-sequencing: SCLC-A (it is characterized by transcription factors expressions such as high ASCL1, high DLL3, and protein Schlafen 11 expression), SCLC-P (it is characterized by transcription factors expressions such as high POU2F3 expression), SCLC-N (it is characterized by transcription factors expressions such as high expression of NEUROD1, high expression of somatostatin receptor 2), and SCLC-Y (it is characterized by YAP1 expression) (Sayed and Blais, 2021). Recent studies have also pointed to the presence of an immune-dependent subtype, referred to as SCLC-I with increased immune infiltration (higher antigen presentation + immune cell infiltration) (Rudin et al., 2019; Gay et al., 2021). These subcategories were divided into those with (SCLC-A, SCLC-N) or without (SCLC-P, SCLC-I) neuroendocrine differentiation. Distinct features and therapeutic vulnerabilities were noted in each sub-category. Therefore CART cell therapy targeting only DLL3 presumably may be effective in an only subcategory of SCLC-A.

## Cancer Stem Cells

### Molecular Characterization of Cancer Stem Cells

The induction of a tumor can be caused by transformed differentiated cells or transformed somatic stem cells (Hanahan and Weinberg, 2011). Transformation of differentiated cells can occur during tissue regeneration and can be initiated and/or accelerated by infection, chronic inflammation, toxins, radiation, or metabolic disorders (Blackadar, 2016; Basu, 2018). During the transformation process, overexpression of oncogenes and inactivation of tumor suppressors are often observed (Hanahan and Weinberg, 2011). As a result, somatic cells de-differentiate and acquire stem character accompanied by uncontrolled growth. Transformation of somatic stem cells or early precursors is caused by a set of genomic changes that allow uncontrolled and niche-independent proliferation (Reya et al., 2001; Li and Neaves, 2006).

In squamous cell carcinomas, the phenotype of cancer cells depends on the source cell and the type of driver mutation responsible for the invasiveness and aggressiveness of the tumor (Yamano et al., 2016; Sánchez-Danés and Blanpain, 2018). Basal stem cells located in the trachea and bronchi are capable of forming heterogeneous spheres. This allows us to consider them as precursors of squamous cell lung cancer (Fukui et al., 2013; Hardavella et al., 2016). It is believed that adenocarcinomas can originate from bronchoalveolar stem cells or pneumocytes of type I and type II (Hardavella et al., 2016). In healthy lungs, these bronchoalveolar stem cells are inactive, but they can begin to actively proliferate and become targets of various factors that cause mutation and transformation (Kim et al., 2005; Hardavella et al., 2016). At an early stage of lung morphogenesis, pulmonary neuroendocrine cells can be identified. In the adult body, after injury from tobacco smoke, oxidative stress, nitrosamines, or burn injury, pulmonary neuroendocrine cell hyperplasia is observed, leading to SCLC, which is a poorly differentiated, high-grade carcinoma (Sutherland et al., 2011; Travis, 2012; Wang et al., 2020).

The drug resistance of SCLC to therapy is associated with tumor heterogeneity. When conducting chemotherapy or radiation therapy, the composition of the tumor changes (Ayob and Ramasamy, 2018; Arnold et al., 2020). In the course of treatment, actively proliferating cancer cells become targets, leading to partial elimination of the tumor (Shibata and Hoque, 2019). At the same time, quiescent cancer stem cells (CSC) survive and may be the main factor in tumor recurrence. Thus, CSCs are potential therapeutic targets, and diagnostic and prognostic markers for SCLC. However, the full potential of these findings has not yet been exploited.

CSC expresses tissue-specific cell surface markers, but none of these markers are exclusive to CSCs. The immunophenotype of CSC may not necessarily be the same for cancer subtypes or even tumors of the same subtype (Klonisch et al., 2008; Rivera et al., 2011). To date, CD133 is the most well documented marker of lung CSC. Lung tumor cells with a high level of CD133 are resistant to chemotherapeutic drugs. Interestingly, expression of CD133 increases even further after the treatment (Sarvi et al., 2014). A high level of expression of CD133 indicates poor outcomes in patients with lung cancer (Rivera et al., 2011; Alamgeer et al., 2013; Huang et al., 2015; Liou, 2019). CD133 is found in a wide range of malignant tumors and characterizes a population of tumor initiating cells in solid breast cancer, as well as in SCLC and NSCLC (Eramo et al., 2008; Tirino et al., 2009). High expression levels of CD133 are also found on undifferentiated cells such as hematopoietic stem cells, endothelial progenitor cells, and neural stem cells (MacDonagh et al., 2017). It was discovered in CD133^+^ SCLC cells, an increased expression of the mitogenic neuropeptide receptors for a gastrin-releasing peptide and arginine-vasopressin (Sarvi et al., 2014). In their study, Zhang Z. et al. found a high degree of stemness, tumorigenicity, and plasticity of NCI-H446 SCLC cells. The markers for these stem cells were CD133, Sall4, Oct4, nestin, neural cell adhesion molecule (NCAM), S100β, vimentin, CD44, and CD105. These cells formed subcutaneous xenograft tumors and orthotopic lung xenograft tumors in BALB/C-nude mice and expressed stem cell markers and the proliferation marker Ki67 (Zhang et al., 2013).

CD44 is another CSC marker and at the same time a cell adhesion molecule usually required for metastasis of CD133^+^ CSC in cancer (Liou, 2019). In addition to CSCs, CD44 is expressed on endothelial and mesenchymal cells (Rivera et al., 2011). Wang P. and colleagues have created a number of lung cancer cell lines from primary tumors. One of the lines was characterized as CD44^high^CD90^+^ and this set of markers has been proposed for the identification of CSC (Asselin-Labat and Filby, 2012). The high level of expression of CD44, which was found in squamous cell carcinomas, correlated with tumors of a higher malignancy (Roudi et al., 2015). CD44v8-10, also known as CD44R1 and CD44E, is one of the isoforms of CD44v and contains variant exons 13-15 (v8-v10) (Ponta et al., 2003; Zöller, 2011). Clinical studies have shown that CD44v8-10 is expressed on a variety of human epithelial malignant tumors, including lung tumors and CSC, and its expression correlates with metastasis (Yamaguchi et al., 1995; Yamaguchi et al., 1996; Okamoto et al., 1998; Olsson et al., 2011; Hiraga and Nakamura, 2016).

Another marker often used for the identification of CSC is aldehyde dehydrogenase (ALDH). ALDH is a superfamily of 19 human isoenzymes and is actively expressed on both healthy cells and cancer cells with stem characteristics (Jones et al., 1995; Ginestier et al., 2007; Huang et al., 2009). Overexpression of ALDH1 is usually associated with a poor prognosis in patients with SCLC (Jiang et al., 2009; Okudela et al., 2013; MacDonagh et al., 2017). Analyzing the activity of CD133 and ALDH, Akunuru S. et al. came to the conclusion that separated tumor stem cells (CD133^+^ ALDH^high^) can mutually transform from non-CSC (CD133^-^ or ALDH^low^) (Akunuru et al., 2012). This process is initiated by TGF-β. The transformation of CSC ↔ non-CSC emphasizes the dynamic plasticity of cancer cells (Hiraga and Nakamura, 2016).

In solid tumors, including lung cancer, overexpression of the plasminogen activator urokinase (uPA) and its receptor (urokinase plasminogen activator receptor, uPAR) known as CD87 has been demonstrated. CD87 is actively involved in the migration and regulation of cell adhesion. High expression of CD87 correlates with unfavorable clinical outcome and significantly shorter overall survival in SCLC (Jones et al., 1995; Ginestier et al., 2007). The population of CD87^+^ cells contains a subpopulation of CSC, has the ability to form spheres and possesses an increased potential for initiating tumor growth, as well as a significant resistance to numerous traditional chemotherapeutic agents intended for the treatment of patients with SCLC (Ginestier et al., 2007; Ginestier et al., 2007; Huang et al., 2009; Jiang et al., 2009; Okudela et al., 2013; MacDonagh et al., 2017). The markers CD87 and CD133 were also identified as markers of CSC in SCLC (Jiang et al., 2009).

CD117 (KIT, c-KIT) is a type III receptor tyrosine kinase that is phosphorylated upon binding of its ligand and stem cell factor (SCF), which leads to activation of multiple signal transduction pathways that regulate a number of biological processes, such as apoptosis, differentiation, adhesion, and cell proliferation (Huang et al., 2009; Walcher et al., 2020). SCF and CD117 are overexpressed in lung cancer (Jiang et al., 2009). High expression of SCF and CD117 in lung cancer is closely associated with smoking status, lower survival rates, and chemoresistance (Kubo et al., 2013; Miettinen and Lasota, 2005; Liou, 2019).

The worst overall survival in SCLC was found if high levels of SOX2 expression were detected in CSCs (Donnenberg et al., 2012; Walcher et al., 2020). Overall, expression of the SOX2 protein is associated with aggressive tumors (Saigusa et al., 2009; Levina et al., 2010; Wilbertz et al., 2011; Perumal et al., 2014; Walcher et al., 2020). Upregulation of SOX2 enhances the proliferation of cancer cells and is important for the function of the lung CSC (Wang et al., 2009; Sholl et al., 2010).

Cripto (TDGF1, CRIPTO-1) is a small glycosylphosphatidylinisitol-secreted oncofetal protein that plays an important role in the regulation of stem cell differentiation, embryogenesis, growth and tissue remodeling (Du et al., 2011). When expressed aberrantly, Cripto can stimulate the onset and progression of various types of tumors, including lung cancer (Nakatsugawa et al., 2011). Cripto regulates signaling pathways involved in cell differentiation and development, such as Wnt and Notch (Xiang et al., 2011). Cripto has been shown to interact with a variety of signaling pathways that play a key role in the regulation of normal tissue homeostasis and tumorigenesis. Adult tissues show low levels of Cripto expression, while increased levels of Cripto *in situ* or in the bloodstream are found in many human cancers (Klauzinska et al., 2014; Xu et al., 2017). Resistance to therapy and high oncogenicity of Cripto^+^ cells are associated with the role of Cripto in maintaining the phenotype of CSC and tumor cells (Du et al., 2011; Nagaoka et al., 2013).

B7-H3 (CD276) is a molecule of the B7 family (Mao et al., 2017; Zhang X. et al., 2017). The B7-H3 protein is found in several types of tumor tissues, including NSCLC and prostate cancer. The molecule is expressed by tumor cells and tumor vascular cells, found in clinical samples of human cancer metastases. B7-H3 has been characterized as a co-stimulatory molecule for T-cell activation. The non-immunological activity of B7-H3 is associated with various signaling pathways, interacting with which changes angiogenesis and tumor invasion. B7-H3 is supposed to be used as a target for cancer treatment and as a marker of the evasion of tumor cells from the action of the immune system (Mao et al., 2017; Zhang Q. et al., 2017; Yang et al., 2020).

AXL belongs to the TAM family (Tyro3, AXL, Mer). GAS6 serves as a ligand for AXL with high binding affinity. GAS6/AXL signaling is an important pathway governing the survival, proliferation, migration, and invasion of tumor cells (Zhu et al., 2019). This makes AXL a potential target for cancer treatment. In adults, AXL expression is relatively low (Vajkoczy et al., 2006), but aberrant Gas6/AXL expression has been detected in a number of human malignancies, including breast cancer, chronic lymphocytic leukemia (CLL), lung cancer, pancreatic cancer, glioblastoma, melanoma. This altered expression is associated with disease progression and reduced overall survival. AXL controls cell proliferation through effector molecules in the PI3K/AKT/mTOR, RAS/RAF/MEK/ERK, JAK/STAT, and NF-κB signaling pathways (May et al., 2015) and correlates with stem cell marker genes such as Isl1, Cdc2a, Bglap1, CD44 and ALDH1 (Asiedu et al., 2014). Expression of marker genes increases the oncogenicity of breast cancer stem cells. It is suggested that targeting AXL has great therapeutic potential and may disrupt Wnt/α-catenin and TGFâR signaling and spherical formation, thereby increasing resistance to cancer and its progression (Yen et al., 2017). Given the role of AXL in the development, progression, and drug resistance of cancer, AXL holds great promise as a predictive biomarker and therapeutic target. Several AXL inhibitors have been found to be promising after preclinical studies and are thus in various stages of clinical trials (Tibes et al., 2013; Rodon et al., 2017; Vergote et al., 2017).

Localization and function of molecular markers of Cancer Stem Cells in Lung are summarized in Table 2.

**TABLE 2:** Markers of lung cancer stem cells.

Marker	Localization, functions
CD133	Member of pentaspan transmembrane glycoproteins, which specifically localizes to cellular protrusions. CD133 is expressed in hematopoietic stem cells, endothelial progenitor cells, glioblastoma, neuronal and glial stem cells, various pediatric brain tumors, as well as in the adult kidney, lung, mammary glands, trachea, salivary glands, uterus, placenta, digestive tract, testes, and some other cell types. CD133 is the most commonly used marker for isolation of CSC population from different tumors, mainly from various gliomas and carcinomas (Eramo et al., 2008; Klonisch et al., 2008; Tirino et al., 2009; Rivera et al., 2011; Alamgeer et al., 2013; Sarvi et al., 2014; Huang et al., 2015; MacDonagh et al., 2017; Liou, 2019).
CD44 CD44v8–10	Cell-adhesion molecule. Expression in normal tissues is low. In addition, CD44 is expressed on endothelial and mesenchymal cells. CD44^+^ cells showed increased self-renewal ability and increased ability to initiate tumor *in vivo* compared to CD44^−^ cells (Yamaguchi et al., 1995; Yamaguchi et al., 1996; Okamoto et al., 1998; Ponta et al., 2003; Olsson et al., 2011; Rivera et al., 2011; Zöller, 2011; Asselin-Labat and Filby, 2012; Roudi et al., 2015; Hiraga and Nakamura, 2016; Liou, 2019).
CD87	Participates in cell migration, regulates cell adhesion. High CD87 expression correlates with poor clinical outcome and significantly shorter overall survival in SCLC. CD87^+^ cell population demonstrated a high spherical ability, an increased tumor initiation potential, and significant resistance to traditional chemotherapeutic agents in SCLC therapy (Romer et al., 2004; Gutova et al., 2007; Almasi et al., 2013; Kubo et al., 2013; Codony-Servat et al., 2016; MacDonagh et al., 2017).
CD117	CD117 (c-KIT) is a type III receptor tyrosine kinase. c-KIT is activated (phosphorylated) by binding of its ligand with stem cell factor (SCF). This leads to activating of signal cascade which activation apoptosis, cell differentiation, proliferation, chemotaxis, and cell adhesion. Overexpression of SCF and CD117 is observed in lung cancer. High SCF expression in lung adenocarcinoma is associated with poor prognosis. Overexpression of CD117 in lung tumors is also associated with poor prognosis, lower survival, and chemoresistance (Romer et al., 2004; Miettinen and Lasota, 2005; Kubo et al., 2013; Liou, 2019; Walcher et al., 2020).
ALDH	High activity of aldehyde dehydrogenase (ALDH) has been found in stem and progenitor cells. High activity and/or overexpression of ALDH can be used as a marker of CSC in various types of cancers, including lung cancer. Overexpression of ALDH1 is associated with a poor prognosis in patients with lung cancer and more severe histological grade and stage of the disease (Jones et al., 1995; Ginestier et al., 2007; Huang et al., 2009; Jiang et al., 2009; Okudela et al., 2013; MacDonagh et al., 2017).
SOX2	There is a relationship between SOX2 expression and SCLC stage and overall survival. SOX2 expression is associated with more aggressive tumors. An increase in SOX2 activity enhances the proliferation of tumor cells. Overexpression of SOX2 is important for lung CSC function (Saigusa et al., 2009; Wang et al., 2009; Levina et al., 2010; Sholl et al., 2010; Wilbertz et al., 2011; Donnenberg et al., 2012; Perumal et al., 2014; Walcher et al., 2020).
CRIPTO-1	There is cell surface glycosylphosphatidylinositol (GPI)-linked glycoprotein which plays an important role in the regulation of stem cell differentiation, embryogenesis, growth and tissue remodeling. Aberrant Cripto expression can stimulate the development and progression of various types of tumors, including lung cancer. Cripto regulates Wnt and Notch signaling pathways. Cripto interacts with a variety of signaling pathways that play a key role in regulating normal tissue homeostasis and tumorigenesis. Cripto expression in adult tissues is low. Tumors show elevated levels of Cripto *in situ* or in the bloodstream. High resistance to chemotherapy and tumorigenicity are associated with Cripto and CSC (Du et al., 2011; Nakatsugawa et al., 2011; Xiang et al., 2011; Nagaoka et al., 2013; Klauzinska et al., 2014; Xu et al., 2017).

### Cancer Stem Cells as a Target for Cancer Therapy

The search and development of cancer treatment options focused on inhibiting, damaging and eliminating CSCs has been the focus of research over the past years (Almasi et al., 2013; Walcher et al., 2020). According to Shibata M. and Hoque M.O., the combination of therapy aimed at SSC and traditional non-targeted therapy can lead to a decrease in chemoresistance (Shibata and Hoque, 2019). Approaches to cancer therapy, targeting CSCs, include urokinase plasminogen activator inhibitors as well as stem cell-related pathways such as Wnt and β-catenin (Almasi et al., 2013; Shibata and Hoque, 2019; Walcher et al., 2020). So far, most of these approaches have failed as front-line treatments including immunological approaches, targeting CSC include adoptive cell transfer, targeting checkpoint inhibitors, and antibody-based approaches and vaccination.

As a result of complete and durable responses in individuals who are refractory to standard of care therapy, CAR T cells directed against the CD19 protein have been granted United States Food and Drug Administration (FDA) approval as a therapy for the treatment of pediatric and young adult acute lymphoblastic leukemia and diffuse large B cell lymphoma. Human trials of CAR T cells targeting CD19 or B cell maturation antigen in multiple myeloma have also reported early successes (Long et al., 2018; Schmidts and Maus, 2018). However, a clear and consistently reproducible demonstration of the clinical efficacy of CAR T cells in the setting of solid tumors has not yet been reported. Despite many difficulties, CART cell therapy for solid cancer can be an effective alternative to chemotherapy and radiation therapy (Long et al., 2018; Schmidts and Maus, 2018; Walcher et al., 2020). In experiments on laboratory animals, a monotherapy of solid cancer against directed to CD133^+^ CTCs showed inhibition of tumor growth in an orthotopic mouse model of glioma (Zhu et al., 2015; Hu et al., 2019). Promising preclinical results have been obtained with combined cell therapy with cytostatic agents (Klapdor et al., 2017). A clinical trial of CD133-targeted CART cells in patients with breast, brain, liver, pancreas, ovarian, and colorectal cancers has been completed (NCT02541370). In patients with previously treated advanced hepatocellular carcinoma, CART-133 cell therapy demonstrated promising antitumor activity and a manageable safety profile.

Several CAR-based approaches have been developed to target CD44. CAR developments in clinical trials include monoclonal antibodies and antibody conjugates. Early studies with 186 Re-conjugated antibodies against the CD44v6 splicing variant showed positive results (Colnot et al., 2002).

EGFR VIII (epidermal growth factor) is rarely expressed in healthy tissues, making this mutated tumor receptor an attractive target for therapeutic molecules. EGFR overexpression is commonly seen in patients with NSCLC. Molecules that inhibit the activity of EGFR kinase show therapeutic effects. The efficacy of second-generation EGFR-specific T-cells that include the signaling domains CD137 and CD3ζ (CD247) has been reported. The anticancer efficacy of EGFR-positive T-cells against lung carcinoma has been demonstrated *in vitro* as cytotoxicity and secretion of interferon γ (IFN-γ) and IL-2. In a phase I clinical trial, two of 11 patients with refractory NSCLC experienced a partial antitumor response after treatment with second-generation EGFR-specific CART cells (Feng et al., 2017; Yang J. et al., 2017).

Mesothelin is a tumor differentiation antigen that is normally present on the mesothelial cells lining the pleura, peritoneum, and pericardium. Since mesothelin is overexpressed in several cancers and is immunogenic, the protein could be exploited as a tumor marker, unfortunately, it is not a specific antigen for cancer (Colnot et al., 2002; Ginestier et al., 2007; Feng et al., 2017; Klapdor et al., 2017; Hu et al., 2019). However, compared with a low level of expression in normal tissues, mesothelin is overexpressed in about 30% of all cancers, including most epithelioid mesotheliomas; adenocarcinomas of the lungs, stomach, bile ducts, endometrium and pancreas; serous ovarian cancer; squamous cell carcinomas of the head and neck, esophagus, lungs, cervix, and vulva; triple negative breast cancer, desmoplastic small cell tumors and epithelial biphasic synovial sarcomas (Chang et al., 1992; Chang and Pastan, 1996; Yang et al., 2017; Hu et al., 2019; Walcher et al., 2020). Numerous preclinical studies have reported the antitumor efficacy of mesothelin and carcinoembryonic antigen-specific T-cells against antigen-positive tumors such as ovarian and liver cancer. However, there is no direct evidence of antitumor efficacy against primary tumor specimens or lung cancer cell lines. CAR meso T-cells are T-cells, which are transduced with a CAR, composed of anti-mesothelin scFv fused to TCRzeta signaling and co-stimulatory domains. ScFv SS1 is derived from a murine monoclonal antibody and is the most studied. In mice, lentiviral (DNA) CART mesocells, which are injected intratumor or intravenously into mice with pre-established tumors, caused a significant reduction in tumor size or destruction of tumors (Carpenito et al., 2009; O’Hara et al., 2016). Positive results of preclinical studies of the antitumor activity of human mesothelin-specific single-chain antibody variable fragment (P4 scFv) coupled to T cell signaling domains were obtained (Lanitis et al., 2012). Importantly, adoptive transfer of P4 CAR-expressing T cells mediated the regression of large, established tumor in the presence of soluble mesothelin in a xenogenic model of human ovarian cancer. This gives hope for the use of CART mesocells in the treatment of patients with mesothelin-expressing tumors.

## Future Perspectives

SCLC accounts for 10–15% of all known cases of lung cancer and is one of the most malignant and incurable tumors. All types of small cell carcinoma have a poor prognosis even when the diagnosis is made at an early stage. Clinical approaches to treating SCLC (surgery, radiation therapy, and chemotherapy) do not lead to patient recovery. High tumor mutational burden (TMB) and high neoantigens production are the main reasons for the progression of treated lung cancer. Until now, CART lymphocytes have not found their application in the treatment of SCLC. This may be due to the prescription of CART lymphocytes when the tumor mass is formed, whereas CART therapy is a more subtle treatment approach than, for example, radiation therapy and chemotherapy, and is designed to target detection and destruction of tumor cells. Thus, there is an urgent need for new approaches to treating SCLC, or revising the tactics of prescribing existing ones.

**FIGURE 2:**
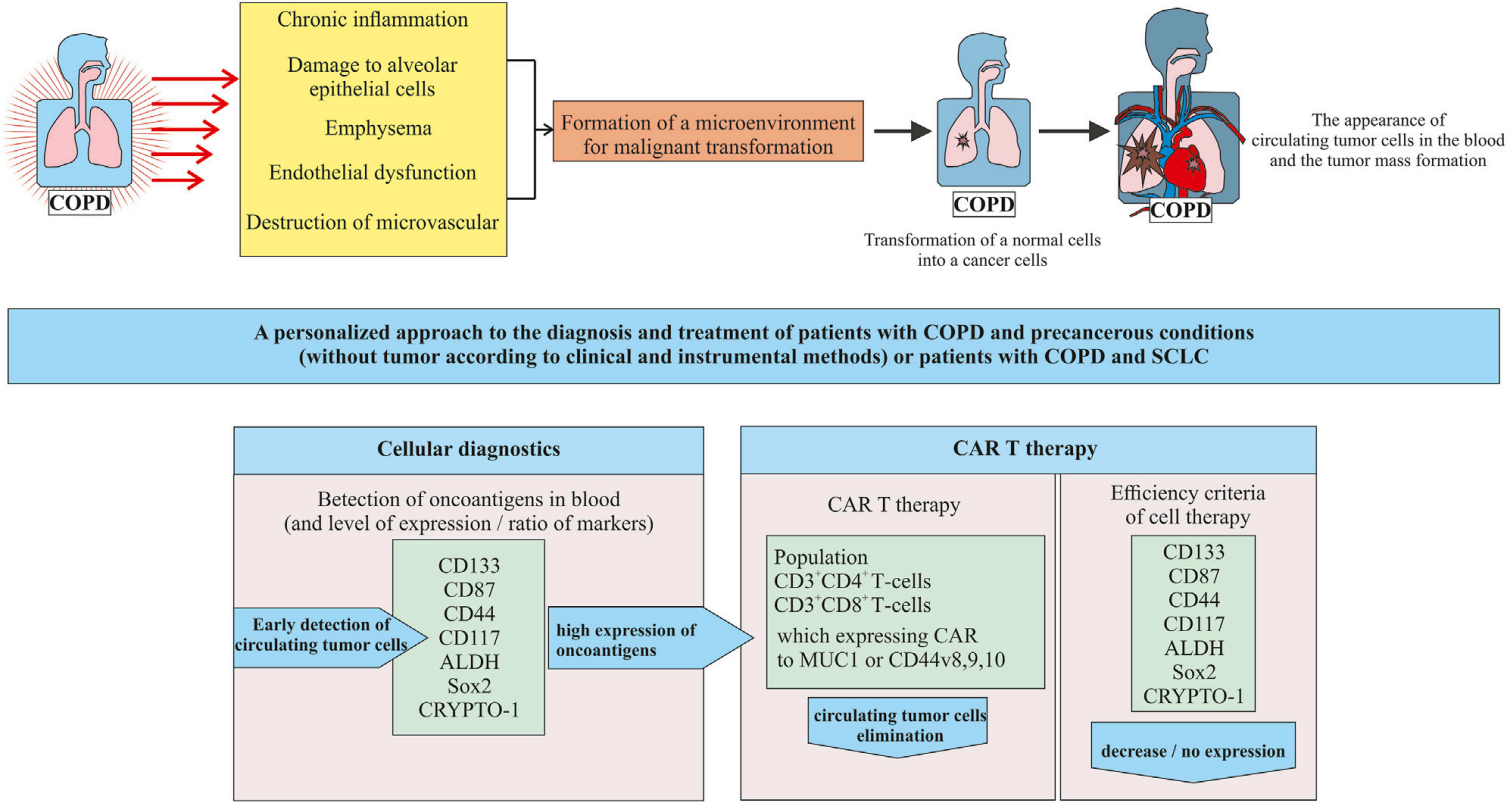
A new approach to cell therapy for patients with COPD in precancerous conditions (tumor mass in the lungs is not yet formed) or patients with COPD and SCLC.

It is known that COPD increases the risk of NSCLC. There is much less data on the link between COPD and SCLC (Ju et al., 2018). Thus we focused on a group of COPD patients who develop SCLC (Figure 2).

In our opinion, in patients with COPD as a precancerous condition, prevention of SCLC at the stage of transformation of healthy cells into tumor cells seems to be promising. As diagnostic and prognostic markers of lung cancer are associated with COPD, it is proposed to assess the level of neutrophils, cholesterol (Mouronte-Roibás et al., 2019), and alpha1-antitrypsin in peripheral blood (Mouronte-Roibás et al., 2019; Tubío-Pérez et al., 2021). A number of studies indicated the possibility of CSCs using as a diagnostic marker of NSCLC in patients with COPD (Ilie et al., 2014; Marquette et al., 2020). There are few studies examining predictors of SCLC (Huang et al., 2018). In a pilot study, we have demonstrated the need to study different populations of CSCs in breast cancer (Pershina et al., 2021). Similarly, detecting circulating CSCs might by the key to early diagnosis of SCLC.

The first question that arises during the transition to this platform is related to the detection of CTCs (including CSC) in the blood of patients in a precancerous condition. At the stage of precancerous condition, we propose to use already known tumor markers as diagnostic markers of CSC including CD133, CD87, CD44, CD117, ALDH, Sox2, and CRYPTO-1. The need to assess the entire spectrum of antigens is justified by the high heterogeneity of the CSC phenotype in SCLC. Compliance with this requirement puts us on a path towards stratification and personalization from the beginning of the detection of CSCs in blood. Not all tumor antigens are expressed or expressed at the same level in all patients. A number of experts in the field are inclined to believe that when choosing a target marker, attention should be paid to antigens with a significant level of expression and to their ratio.

The appearance of one or a group of tumor markers in the blood of patients with a precancerous condition is a signal for CART therapy. To create CARs, populations of CD3^+^CD4^+^ and CD3^+^CD8^+^ blood T cells are proposed. These cells have proven themselves well as the basis for CARs aimed at other conditions. We further propose to use the expressed tumor markers identified in the patient as targets for modified T cells.

Importantly, potentially negative effects of the therapy need to be considered. To reduce the side effects risk of the CART therapy and to increase the selectivity of CART cells in relation to ROS, specific antigens such as EGFRvIII and CD44v8-10 are proposed in addition to the already identified antigens. Creation of CART cells capable of recognizing multiple antigens can significantly increase the effectiveness of CART therapy. A promising approach to the design of CART cells functioning according to the “AND” gate, which requires the simultaneous activation of two receptors for the manifestation of an immune response. With a positive outcome of CART therapy in patients with a precancerous condition, the need for chemotherapy and radiation therapy may disappear. One of the approaches to increase the effectiveness of SCLC treatment is the modulation of immune response and effect on the tumor microenvironment activity.

We propose a new approach for an increase of CART therapy efficiency in SCLC. However, our review and the proposed strategy have some limitations. To assess their applicability to a clinic, the results discussed here need further validation in pre-clinical studies and multi-center clinical studies focused on cohorts with large numbers of patients.

## Abbreviations

CAR, Chimeric antigen receptor; CART therapy, Chimeric antigen receptor T cells therapy; COPD, Chronic obstructive pulmonary disease; CSC, Cancer stem cells; MAB, Monoclonal antibodies; NSCLC, Non-small cell lung cancer; SCLC, Small cell lung cancer.
